# Chordin-mediated BMP shuttling patterns the secondary body axis in a cnidarian

**DOI:** 10.1126/sciadv.adu6347

**Published:** 2025-06-13

**Authors:** David Mörsdorf, Maria Mandela Prünster, Paul Knabl, Grigory Genikhovich

**Affiliations:** ^1^ Department of Neurosciences and Developmental Biology, University of Vienna, Djerassiplatz 1, 1030 Vienna, Austria.; ^2^ Vienna Doctoral School of Ecology and Evolution (VDSEE), University of Vienna, Vienna, Austria.

## Abstract

Bone morphogenetic protein (BMP) signaling patterns secondary body axes throughout Bilateria and in the bilaterally symmetric corals and sea anemones. Chordin-mediated “shuttling” of BMP ligands is responsible for the BMP signaling gradient formation in many bilaterians and, possibly, also in the sea anemone *Nematostella*, making BMP shuttling a candidate ancestral mechanism for generating bilaterality. However, *Nematostella* Chordin might be a local inhibitor of BMP rather than a shuttle. To choose between these options, we tested whether extracellular mobility of Chordin, a hallmark of shuttling but dispensable for local inhibition, is required for patterning in *Nematostella*. By generating localized Chordin sources in the Chordin morphant background, we showed that mobile Chordin is necessary and sufficient to establish a peak of BMP signaling opposite to Chordin source. These results provide evidence for BMP shuttling in a bilaterally symmetric cnidarian and suggest that BMP shuttling may have been functional in the potentially bilaterally symmetric cnidarian-bilaterian ancestor.

## Teaser

Chordin-mediated BMP shuttling is the candidate mechanism for generating bilateral symmetry in the cnidarian-bilaterian ancestor.

## INTRODUCTION

Bone morphogenetic protein (BMP) signaling acts in secondary body axis patterning across Bilateria, and its functions as morphogen have been studied in diverse animal species (*1*, *2*). The mechanisms of the BMP-dependent axial patterning are similar between arthropods and vertebrates, indicative of the shared origin of the secondary, dorsoventral axis in protostome and deuterostome Bilateria, a notion strengthened once broader phylogenetic sampling became available (*2*–*7*). Intriguingly, the same mechanisms appear to regulate the secondary axis patterning in the bilaterally symmetric cnidarian *Nematostella vectensis*, indicating that a BMP-dependent secondary body axis may have evolved before the evolutionary split of Cnidaria and Bilateria [(*8*, *9*), reviewed in (*1*, *10*)]. However, a scenario in which BMP-mediated secondary axes evolved convergently in Bilateria and bilaterally symmetric Cnidaria is also possible (*2*).

**Fig. 1.**
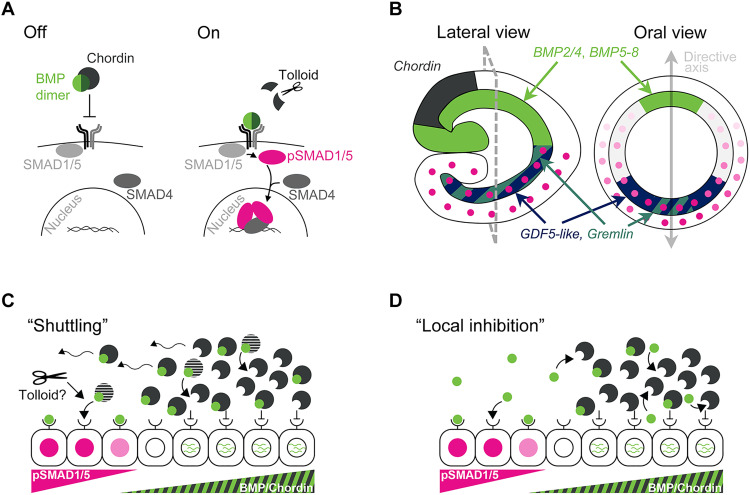
Possible modes of action of BMP signaling during axial patterning in *Nematostella*. (**A**) BMP signaling pathway. BMP dimers bind the heterotetrameric receptor complex, resulting in the phosphorylation of SMAD1/5. pSMAD1/5 forms a complex with the Co-Smad SMAD4, which regulates transcription in the nucleus. Chordin binds BMPs preventing them from activating the receptor complex. Metalloproteases like Tolloid and BMP-1 cleave Chordin and release BMP ligands from the inhibitory complex in Bilateria. (**B**) Expression domains of BMPs and BMP antagonists in an early *Nematostella* larva. Oral view corresponds to the optical section indicated with grey dashed line on the lateral view. Pink circles show the nuclear pSMAD1/5 gradient. (**C**) The shuttling model suggests that in *Nematostella*, a mobile BMP-Chordin complex transports BMPs through the embryo. Receptor binding is inhibited in cells close to the Chordin source due to high concentrations of Chordin. On the opposite side of the directive axis, BMPs bind their receptors and activate signaling upon release from Chordin. Tolloid might be involved in the cleavage of Chordin and release of BMPs from the complex with Chordin also in *Nematostella*. (**D**) In the local inhibition model, *Nematostella* Chordin acts locally to inhibit BMP signaling and promote the production of *BMP2/4* and *BMP5-8* mRNA. Chordin mobility is not required for asymmetric BMP signaling.

BMPs are secreted signaling proteins of the transforming growth factor–β superfamily frequently acting as heterodimers (*11*–*13*). Signaling through the BMP receptor complex (Fig. 1A) results in phosphorylation and nuclear accumulation of the transcriptional effector SMAD1/5, which regulates the expression of many crucial developmental transcription factors and signaling pathway components [(*14*–*18*), reviewed in (*19*, *20*)]. BMP signaling is tightly controlled by a plethora of intracellular (*14*, *21*) and extracellular regulators (*22*–*29*) of which Chordin (= short gastrulation in insects) is, arguably, the most famous one. Like many other secreted BMP antagonists, Chordin binds BMP ligands, blocks the interaction with their receptor, and thereby inhibits BMP signaling (*30*). However, Chordin can also have pro-BMP effects and promotes long-range activation of BMP signaling in *Drosophila*, *Xenopus*, sea urchins, and in the sea anemone *Nematostella* (*7*, *31*–*34*). The phylogenetic distribution of Chordin and two central BMP ligands, BMP2/4 and BMP5-8, and their importance for the secondary axis patterning across phyla suggests that, during early animal evolution, these molecules may have represented the minimum requirement for the formation of the bilaterally symmetric body plan (*2*, *10*). However, to evaluate such a possibility, we need to understand the “mode of action” of BMPs and Chordin outside Bilateria, and our model, the sea anemone *Nematostella*, allows exactly that.

BMP signaling in *Nematostella* becomes detectable during early gastrula stage in a radially symmetric domain: The phosphorylated form of the BMP signaling effector SMAD1/5 (pSMAD1/5) is detected in the nuclei around the blastopore (*14*, *35*). Shortly after the onset of BMP activity, the radial symmetry of the embryo breaks, establishing the secondary, “directive” body axis with minimum BMP signaling intensity detectable on the side of *BMP2/4*, *BMP5-8*, and *Chordin* expression and maximum BMP signaling on the side opposite to it (Fig. 1B) (*14*, *34*, *35*). The symmetry break occurs despite the fact that mRNAs of the type I BMP receptors Alk2 and Alk3/6 and the type II receptor BMPRII are maternally deposited (*36*) and remain weakly and ubiquitously expressed in the embryo (fig. S1) gradually developing a slight bias toward the “high pSMAD1/5” side of the directive axis by early planula stage (*14*). *BMP2/4* and *BMP5-8* are co-expressed in the late gastrula/early planula, and both these ligands are crucial for BMP signaling and directive axis patterning because knockdown of either ligand abolishes pSMAD1/5 immunoreactivity and completely radializes the embryo (*34*). Individual knockdowns of either BMP2/4 or BMP5-8 result in a strong up-regulation of transcription of both *BMP2/4* and *BMP5-8* in a radially symmetric domain showing that both these genes are negatively controlled by BMP signaling. Despite transcriptional up-regulation of *BMP2/4* in BMP5-8 morphants and *BMP5-8* in the BMP2/4 morphants, no nuclear pSMAD1/5 is observed in such embryos (*9*, *34*, *35*), suggesting that BMP2/4 and BMP5-8 signal as an obligate heterodimer during axial patterning in *Nematostella*.

The “core” BMPs, BMP2/4 and BMP5-8, are not the only BMP ligands present in the embryo at this stage. GDF5-like (GDF5L) is a BMP ligand expressed on the side of strong BMP signaling (Fig. 1B). *GDF5L* expression is abolished in the absence of BMP2/4 and BMP5-8, and the role of GDF5L appears to be in steepening the pSMAD1/5 gradient making it a “modulator” BMP (*14*, *34*, *37*). The BMP signaling gradient is stable over many (>24) hours during which it patterns the directive axis (*9*, *14*, *34*, *35*, *37*). Considering the short half-life of phosphorylated SMAD1/5 reported in other systems (*15*, *21*), this indicates that long-range transport (~100 μm) of BMP2/4 and BMP5-8 and constant receptor complex activation is necessary to maintain BMP signaling. How it exactly happens that the core BMP ligands, BMP2/4 and BMP5-8, are expressed on one side of the embryo and the peak of BMP signaling activity is on the opposite side is currently unknown.

One possible explanation involves Chordin-mediated shuttling of BMP ligands, described in the dorsoventral patterning in *Drosophila* and *Xenopus* (*7*, *34*, *38*). In this model, Chordin inhibits BMP function locally, close to the Chordin source cells, but promotes long-range BMP signaling by forming a mobile complex with the BMP dimer, which is released once Chordin is cleaved by the metalloprotease Tolloid. The probability that this BMP dimer will bind its receptors rather than another, yet uncleaved Chordin increases with the distance to the Chordin source (Fig. 1C). In *Nematostella*, the shuttling model was proposed when we found that, unlike in all bilaterian models studied thus far, depletion of Chordin results in the loss of BMP signaling rather than in its enhancement (*34*). However, given that, in *Nematostella*, BMP signaling indirectly represses the transcription of the core BMPs, *BMP2/4* and *BMP5-8*, and activates the transcription of the modulator BMP, *GDF5-like* (*14*), an alternative explanation is also possible: In this “local inhibition” scenario, Chordin locally represses BMP signaling enabling BMP2/4 and BMP5-8 production. BMP2/4 and BMP5-8 diffuse into the area of low or no Chordin (i.e., to the *GDF5-like* side of the directive axis) and bind the receptors there. In this scenario, Chordin knockdown results in a transient de-repression of the BMP2/4/BMP5-8–mediated signaling, which, in turn, leads to the repression of the *BMP2/4* and *BMP5-8* transcription. Because, in the absence of BMP2/4 and BMP5-8, *GDF5-like* expression is also lost (*9*), we may end in a situation when no BMP ligands are produced and no BMP signaling takes place, as it is the case in the Chordin morphant (*9*, *34*). This local inhibition model, in which Chordin acts exclusively as a local repressor of BMP signaling (Fig. 1D), is similar to the situation in zebrafish, where extracellular mobility of Chordin is not required (*39*–*41*). Here, we address the role of Chordin in the BMP-dependent axial patterning in the sea anemone *Nematostella* and test these two alternative models.

## RESULTS

### BMPs are retained on the surface of the cells and interact with Chordin

**Fig. 2.**
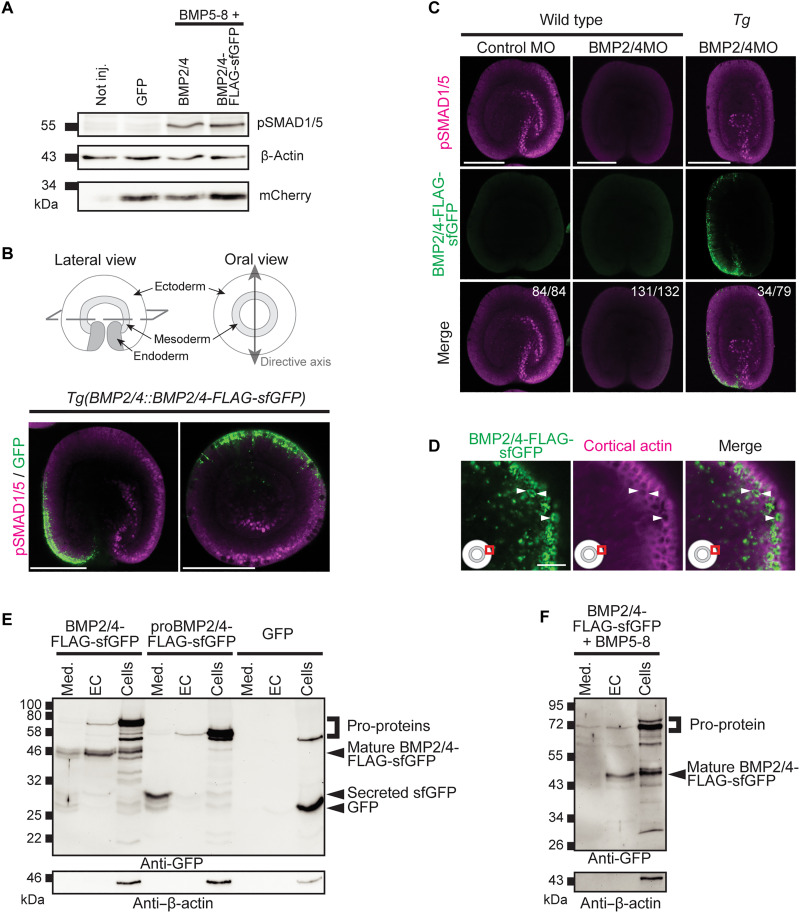
Active *Nematostella* BMP2/4 is retained in association with the cell surface. (**A**) Anti-pSMAD1/5 Western blot confirms that BMP2/4-FLAG-sfGFP can activate BMP signaling, likely as a heterodimer with BMP5-8. *GFP* mRNA was injected at equimolar concentration to the BMP mRNAs and *mCherry-CAAX* mRNA was injected at a fixed concentration in all samples as a reference. *n* = 3. (**B**) Sketches depicting the morphology of a *Nematostella* embryo, lateral and oral views of a 2-dpf *BMP2/4::BMP2/4-FLAG-sfGFP* embryo showing pSMAD1/5 immunofluorescence (magenta) opposite to the BMP2/4 source (green). *n* = 3. (**C**) Anti-pSMAD1/5 immunofluorescence shows that GFP-positive *BMP2/4::BMP2/4-FLAG-sfGFP* embryos retain asymmetric BMP signaling upon injection of BMP2/4MO in 43% of the embryos. Numbers in the top right corner show the fraction of the embryos demonstrating the phenotype shown on the representative image. Lateral views, oral end points down. *n* = 3. (**D**) Confocal image of an area (red frame on the pictogram) in the ectoderm of a fixed, GFP-positive *BMP2/4::BMP2/4-FLAG-sfGFP* embryo shows BMP2/4-FLAG-sfGFP signal inside the cells (white arrowheads), close to their apical or apico-lateral surfaces. At this magnification, the basal surfaces of the ectodermal cells are outside the lower left corner of the imaged area. (**E** and **F**) Western blot of medium (Med.), extracellular (EC), and cellular (Cells) protein fractions from embryos injected with either *BMP2/4-FLAG-sfGFP*, *proBMP2/4-FLAG-sfGFP* (control construct lacking BMP2/4 ligand domain), or *GFP* mRNA. The BMP pro-proteins are detected mostly in the cellular fraction, whereas the mature BMP2/4-FLAG-sfGFP ligand is enriched in the EC fraction. The secreted FLAG-sfGFP is detected almost exclusively in the medium. (F) Mature BMP2/4-FLAG-sfGFP ligand is retained in the EC fraction also when co-injected together with *BMP5-8* mRNA. *n* = 3. Scale bars, [(B) and (C)] 100 μm and (D) 10 μm.

At the time of the BMP signaling gradient formation, the *Nematostella* embryo has two cell layers separated by a thin extracellular matrix, the mesoglea. To get a better understanding of where BMP diffusion takes place, we set out to generate biologically active, detectable BMP2/4, express it in the endogenous domain, and address its distribution. A critical step of the posttranslational processing of BMP ligands is the proteolytic cleavage of the BMP propeptides, separating the pro-domain from the mature domain (*20*). Therefore, we generated a tagged BMP2/4, in which a FLAG–superfolder green fluorescent protein (sfGFP) tag is fused to the N terminus of the mature ligand domain (MLD; fig. S2A). This design is based on previous BMP fusion proteins (*39*) and results in a BMP ligand that can activate BMP signaling at similar levels as the untagged BMP2/4, as shown by pSMAD1/5 Western blot (Fig. 2A and fig. S2B). Co-injection of *BMP2/4* and *BMP5-8* mRNAs leads to a stronger phosphorylation of SMAD1/5 than does the injection of individual ligand mRNAs at 2× concentration (fig. S2, C and D), indicating that BMP2/4/BMP5-8 heterodimers are the biologically relevant BMP ligands in vivo in early embryos, consistent with their identical knockdown phenotypes (*9*, *34*).

Once we confirmed that *BMP2/4-FLAG-sfGFP* was active, we generated a transgenic line in which it is expressed under control of the 4.5-kb DNA fragment upstream of the start codon of *Nematostella BMP2/4* (*42*). An F0 male with germline transmission of the transgene was crossed to a wild-type (WT) female, and pSMAD1/5-positive nuclei were detected by immunofluorescence in sfGFP–positive F1 embryos to visualize the BMP2/4-FLAG-sfGFP protein and the BMP signaling domain simultaneously. Consistent with the previously described expression of endogenous *Nematostella BMP2/4* on the “low–BMP signaling” side of the secondary body axis (*34*), we observed a graded GFP signal with the maximum on the pSMAD1/5-negative side of the 2-day-postfertilization (dpf) embryo (Fig. 2B). Due to several silent mutations, the translation of the BMP2/4-FLAG-sfGFP is not affected by the previously characterized BMP2/4 morpholino [BMP2/4MO; (*9*, *14*, *34*, *43*)], which allowed us to test the activity of the *BMP2/4-FLAG-sfGFP* transgene in the absence of endogenous BMP2/4. The transgene only partially rescued the phenotype of BMP2/4MO-injected embryos, and none of them formed primary polyps, suggesting that some necessary enhancer elements were missing in our construct. However, pSMAD1/5-positive cells could be detected on one side of the directive axis in ~43% of the transgenic BMP2/4MO-injected embryos (34 of 79; Fig. 2C), while non-transgenic BMP2/4MO-injected controls remained pSMAD1/5-negative (131 of 132; Fig. 2C). Thus, we confirmed that BMP2/4-FLAG-sfGFP expressed from a transgene in the endogenous domain has, at least, to some degree, the axis-generating activity of the endogenous BMP2/4, and we proceeded with the analysis of the localization of the BMP2/4-FLAG-sfGFP protein.

In the transgenic line, strong sfGFP signal was detected in clusters at the surface of live embryos as well as fixed embryos, indicating that BMP2/4 ligands are likely to be secreted toward the apical or apico-lateral sides of the producing cells (Fig. 2D and fig. S2E). No extracellular BMP2/4-FLAG-sfGFP was detectable in fixed or live embryos, suggesting that the clusters of BMP2/4-FLAG-sfGFP signal were inside the producing cells, while mature, secreted BMP2/4-FLAG-sfGFP escaped detection with our methods. Because this precluded our initial plan of analyzing BMP transport, we turned to biochemistry to obtain at least some information about the localization of the active BMP2/4 ligands. We established a fractionation protocol allowing detection of sfGFP-tagged BMP2/4 in the cells, on the cell surface, and in the medium by Western blotting with an anti-GFP antibody (fig. S3A). As expected, and in line with our confocal imaging data (Fig. 2D and fig. S2E), the bulk of the signal is observed in the cellular fraction and corresponds to unprocessed BMP2/4-FLAG-sfGFP (“Cells” in Fig. 2E). While mature BMP ligands are secreted, they appear to remain associated with the cell surface and are mainly detected in the extracellular fraction (“EC” in Fig. 2E). In contrast, a secreted sfGFP control containing the BMP2/4 pro-domain followed by sfGFP and lacking the BMP2/4 ligand domain is predominantly secreted into the medium (“Med” in Fig. 2E), while cytoplasmic GFP remains inside the cells. This shows that the mature BMP2/4 ligand domain promotes retention of BMP2/4-FLAG-sfGFP on the cell surface and suggests interactions with the extracellular matrix components or BMP receptor complexes. Similar results are obtained when BMP5-8 is co-expressed with the BMP2/4 constructs (Fig. 2F). As expected, co-immunoprecipitation (CoIP) analysis on embryos injected with a combination of either *BMP2/4-FLAG-sfGFP* or control (*GFP*) mRNA together with either *BMP2/4-mCherry* or *BMP5-8–mCherry* mRNA showed the presence of the BMP2/4/BMP5-8 heterodimer in the extracellular fraction (fig. S3, B and C).

Having established that mature BMP2/4 localizes to the extracellular fraction, we tested by CoIP whether it interacts there with Chordin. We tagged *Nematostella* Chordin C-terminally with FLAG-sfGFP and replaced FLAG-sfGFP in our BMP2/4 construct used for transgenics with the mCherry sequence. Then, we co-injected mRNAs of *BMP2/4-mCherry*, untagged *BMP5-8*, and either *GFP* or *Chd-FLAG-sfGFP* and performed a pulldown using GFP-Trap Magnetic Agarose. We could show that mCherry-tagged BMP2/4 co-precipitated with Chd-FLAG-sfGFP in the extracellular fraction (fig. S3, D and E). In a similar experiment, in which we exchanged mRNAs of the “core BMPs,” BMP2/4 and BMP5-8, with the mRNA of the “modulator BMP,” GDF5-like, Chd-FLAG-sfGFP pulled down GDF5-like–mCherry from the extracellular fraction as well (fig. S3, F and G).

### Long-range BMP signaling requires diffusible Chordin

The main difference between the local inhibition model and the shuttling model is the necessity of Chordin mobility for Chordin-mediated BMP shuttling (*32*, *38*–*41*). As we were unable to detect extracellular BMP2/4-FLAG-sfGFP signal by live imaging and directly measure BMP mobility in the presence and absence of Chordin, we devised an alternative approach. We exploited the fact that no pSMAD1/5 is detectable in Chordin morphants (*34*, *35*) to address the effect of Chordin on BMP signaling and test the requirement for extracellular Chordin mobility. We reasoned that we could generate a localized source of either wild-type (WT) Chordin or immobile, membrane-tethered Chordin-CD2 (*32*, *41*) in the Chordin morphant background and use pSMAD1/5 staining as a readout of the BMP signaling activity.

**Fig. 3.**
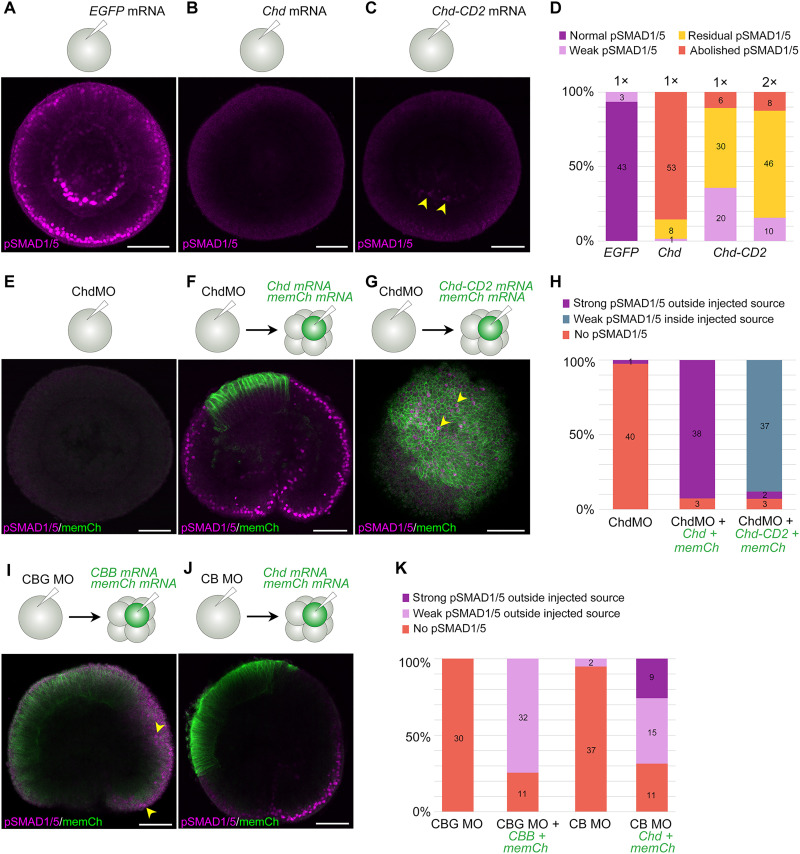
Local source experiments show that mobile Chordin shuttles BMPs. (**A** to **D**) pSMAD1/5 immunofluorescence after injection of *GFP* (A), *Chordin* (B), and *Chordin-CD2* (C) mRNAs shows that Chordin and immobile Chordin-CD2 repress BMP signaling, although residual pSMAD1/5 signal remains in a large fraction of embryos injected with Chordin-CD2 mRNA [yellow arrowheads in (C)] even when the mRNA concentration is doubled [2× in (D)]. *n* = 3. (**E** to **H**) Chordin is required for BMP signaling. (E) pSMAD1/5 immunofluorescence shows absence of BMP signaling when ChdMO is injected at the one-cell stage. *n* > 10. (F) A local source of Chordin generated by single-blastomere mRNA injection into a Chordin morphant results in strong BMP signaling (pSMAD1/5 immunofluorescence) outside of the source cells labeled by mCherry-CAAX expression (memCh). *n* = 3. (G) Same experiment with Chordin-CD2 results in weak BMP signaling in the source cells (yellow arrowheads). *n* = 3. (**I** to **K**) Single blastomere injections followed by pSMAD1/5 immunofluorescence show that a source of Chordin + BMP2/4 + BMP5-8 (CBB) can activate BMP signaling at a distance in embryos that were injected with morpholinos against *Chordin*, *BMP2/4*, and *GDF5-like* [CBG MO in (I), yellow arrowheads indicate pSMAD1/5-positive nuclei]. GDF5-like is, therefore, not required to activate BMP signaling in non-source cells. In embryos injected with ChdMO + BMP2/4MO (CB MO), a local source of Chordin is sufficient to trigger BMP signaling outside of the source, indicating that Chordin promotes also GDF5-like–mediated signaling (J). *n* = 3. See fig. S5 for [(F), (G), (I), and (J)] without a green channel. Numbers overlaid on the bar charts on [(D), (H), and (K)] indicate the number of embryos in each category. Scale bars, 50 μm.

First, we confirmed by anti-GFP CoIP that both Chordin-FLAG-sfGFP and Chordin-FLAG-sfGFP-CD2 were capable of binding BMP2/4-mCherry (fig. S3, C and D). Then, we tested the biological activity of Chordin and Chordin-CD2 by injecting mRNA into zygotes and analyzing BMP signaling and *BMP2/4* expression in late gastrula stage embryos (Fig. 3, A and D, and fig. S4A). As expected, WT *Chordin* mRNA injection abolished the pSMAD1/5 gradient and radialized the expression of *BMP2/4* (Fig. 3, A and B, and fig. S4A). In contrast, bilateral symmetry was not abolished upon injection of the *Chordin-CD2* mRNA; however, pSMAD1/5 intensity was strongly reduced (Fig. 3C and fig. S4A). Reduced anti-BMP activity of Chordin-CD2 was not due to it having a bulky tag at the C terminus. Chordin tagged C-terminally with FLAG-sfGFP was capable of radializing *BMP2/4* expression nearly as efficiently as the WT Chordin; however, when we tethered such Chordin-FLAG-sfGFP to the membrane by adding the CD2 sequence to the C terminus of sfGFP, the injected embryos developed bilateral symmetry (fig. S4A). We concluded that both Chordin and Chordin-CD2 were functional, and the difference in the Chordin and Chordin-CD2 overexpression phenotypes was possibly due to the presence of endogenous Chordin, which was incompletely outcompeted by Chordin-CD2. Therefore, we moved on and used Chordin-CD2 in the localized source experiments.

Previously, we showed that the expression of all three *Nematostella* BMP ligand genes active in the early embryo—*BMP2/4*, *BMP5-8*, and *GDF5-like*—is nearly or completely abolished at 2 dpf upon Chordin knockdown (*34*). However, analysis at 1 dpf (late gastrula) showed that, at this stage, all these genes were still active, although their expression was radialized (fig. S4B). Thus, we concluded that BMP ligands are present in 1 dpf Chordin morphants and analyzed BMP signaling activity in 1 dpf Chordin morphants injected into a single cell at the eight-cell stage with mRNAs of either WT Chordin or membrane-tethered, immobile Chordin-CD2 (*Chd* and *Chd-CD2* in Fig. 3, respectively). To label the source of Chordin, *mCherry-CAAX* mRNA was co-injected with the *Chordin* mRNAs (Fig. 3, E to H). Chordin morpholino injection (ChdMO in Fig. 3) abolished BMP signaling in late gastrula (1 dpf) despite the presence of the BMP ligands (Fig. 3E), suggesting that BMP signaling in *Nematostella* by any type of ligand is Chordin-dependent at this stage. In contrast, creating a local mCherry-CAAX–labeled source of either Chordin or Chordin-CD2 rescued BMP signaling in Chordin morphants, however, in a notably different manner. In Chordin morphants with a local source of WT Chordin, we observed strongly stained pSMAD1/5-positive nuclei outside the Chordin source (Fig. 3F and fig. S5), while, in Chordin morphants with a local source of Chordin-CD2, we saw weakly stained pSMAD1/5-positive nuclei inside the Chordin-CD2 source (Fig. 3G and fig. S5). Moreover, upon simultaneous morpholino knockdown of BMP2/4, GDF5-like, and Chordin (CBG MO in Fig. 3, I and K), which abolishes not only Chordin but also the endogenous BMPs, a local source of Chordin, BMP2/4, and BMP5-8 (CBB mRNA in Fig. 3, I and K) was sufficient to rescue BMP signaling in ~74% of the embryos (Fig. 3, I and K, and figs. S4C and S5). This shows that embryos, in which BMP2/4 and BMP5-8 are only co-expressed in the source cells and GDF5-like is suppressed, are capable of activating BMP signaling in the cells outside the source. Last, we tested whether Chordin was capable of promoting only BMP2/4/BMP5-8–mediated signaling or whether it could do the same with GDF5-like–mediated signaling, which would be in line with our CoIP results (fig. S3, F and G). *GDF5-like* mRNA is present in 1-dpf embryos both upon *Chd* and *BMP2/4* knockdown (fig. S4B); therefore, we injected zygotes with a mixture of Chordin and BMP2/4 morpholinos without the GDF5-like morpholino (CB MO in Fig. 3, J and K) and created local sources of Chordin but did not introduce any exogenous BMPs. In such embryos, BMP signaling on the opposite side to the Chordin source was rescued in ~69% of the cases; moreover, in about 26% of the cases, BMP signaling was strong (Fig. 3, J and K, and figs. S4C and S5). In summary, we conclude that extracellular Chordin mobility is required for long-range activation of BMP signaling and that the agonistic action of Chordin and BMP is not selective with regard to the type of BMP ligand.

## DISCUSSION

Chordin-dependent BMP signaling is required to establish and pattern the second, directive body axis in the bilaterally symmetric cnidarian, the sea anemone *N. vectensis* (*34*, *35*). However, although BMP shuttling has been suggested to be the mechanism underlying this process (*34*), a local inhibition model was a valid alternative. Here, we attempted to learn more about the molecular mechanism of *Nematostella* directive axis formation and the role of Chordin in it.

### BMP heterodimer transport appears to occur in an unexpected location

Our current understanding of BMP signaling in *Nematostella* directive axis patterning is based on a model, in which BMP heterodimers containing BMP2/4 and BMP5-8 diffuse through the mesoglea, a layer of extracellular matrix separating the outer ectodermal layer from the inner endoderm and mesoderm (*34*). We showed that injection of a mixture of *BMP2/4* and *BMP5-8* mRNAs elicits a stronger pSMAD1/5 signal than overexpression of these ligands individually and that BMP2/4/BMP5-8 complexes can be pulled down by CoIP. This is consistent with earlier loss-of-function data (*9*, *34*, *35*) and suggests that BMP2/4/BMP5-8 heterodimers are the biologically relevant BMP ligands during axis patterning in *Nematostella*. This is similar to the situation in Bilateria, where BMP heterodimers outperform homodimers in various developmental contexts (*11*–*13*). However, we still do not understand where BMP diffusion and signaling takes place. Although our attempts to visualize extracellular sfGFP-tagged BMP2/4 failed, we made two important observations. First, the visualization of the intracellular sfGFP-tagged BMP2/4 suggested that it was secreted toward the apical or apico-lateral surface of the ectodermal cells of the *Nematostella* gastrula rather than basally, toward the mesoglea. Second, in our fractionation experiments, we showed that mature BMP ligands were retained on the surface of the cells rather than released into the medium.

The potentially apical or apico-lateral secretion of BMPs is consistent with a previously observed apico-lateral localization of the ectopically expressed GFP-tagged constitutively active type I BMP receptor Alk3/6 (*34*). Secretion toward the outside of the embryo, if experimentally confirmed in future studies, would also not be a unique feature of *Nematostella*. In *Drosophila*, BMP2/4 also appears to be secreted toward the surface of the embryo, and shuttling is thought to happen in the perivitelline space, a sealed-off extraembryonic compartment (*31*). However, *Nematostella* embryos are not surrounded by any extraembryonic membrane, and the retention of BMP ligands in the EC fraction observed in our fractionation experiments may be necessary to prevent the loss of the signaling molecules into the medium and facilitate gradient formation (*44*). Clearly, the assumptions of our 2015 model (*34*) about the geometry of the BMP diffusion and the BMP signaling domain will need to be revisited once we know more about the distribution of mature BMP ligands and receptors in the embryo and the mechanism of BMP retention on the cell surface. One candidate family of potential regulators of the extracellular BMP retention and transport are the heparan sulfate proteoglycans (HSPGs). HSPGs regulate a number of signaling pathways in other models, including BMP signaling, and may act similarly in *Nematostella*. However, to date, the only study investigating *Nematostella* HSPGs found that Glypican 1/2/4/6 and glycosaminoglycan sulfation act primarily on oral-aboral patterning (*45*).

### BMP shuttling as a candidate ancestral mechanism of second axis patterning

BMP-dependent axial patterning systems, although extremely diverse in different animal groups [for review, see (*2*)], tend to repeatedly evolve a “seesaw” regulatory architecture with both ends of the second body axis expressing different BMP ligands and BMP signaling occurring on one of these two ends, opposite to the Chordin expression domain. There are exceptions, especially in insects, where BMPs signal dorsally and Toll signaling plays a role as a ventral signal (*3*, *46*–*50*); however, in anthozoan Cnidaria, such as *Nematostella*, and in Deuterostomia, this seems to be the general rule. For example, in the frog *Xenopus*, the “ventral signaling center” expresses BMP4 and BMP7; the “dorsal signaling center” expresses BMP2, ADMP (another BMP ligand), and Chordin; and the pSMAD1/5 gradient has a ventral maximum (*51*). In the sea urchin *Paracentrotus*, the ventral signaling center expresses BMP2/4, ADMP1, and Chordin; the dorsal signaling center expresses ADMP2; and the pSMAD1/5 gradient has a dorsal maximum (*33*, *52*). In both situations, Chordin-mediated BMP shuttling has been suggested as a patterning mechanism (*7*, *33*, *51*). Moreover, recent data from the brachiopod *Lingula*, with dorsally expressed BMP2/4 and BMP5/8, ventrally expressed ADMP, BMP3, and Chordin, and a dorsal pSMAD1/5 maximum also suggest a BMP seesaw in a spiralian protostome (*53*). The expression of BMP2/4, BMP5-8, and Chordin on one side of the directive axis and of GDF5-like on the other side of the directive axis, and a pSMAD1/5 gradient maximum on the GDF5-like–expressing side in the embryos of the cnidarian *Nematostella* fits neatly to the seesaw paradigm.

One unique feature of the *Nematostella* BMP-dependent axial patterning is the extent to which it relies on Chordin in 1- to 2-dpf embryos. In all bilaterian models, where this has been addressed, Chordin loss of function de-represses BMP signaling expanding the pSMAD1/5-positive domain (*39*, *40*, *51*, *52*, *54*, *55*). In contrast, Chordin knockdown leads to the disappearance of the pSMAD1/5-positive domain in *Nematostella*, despite the presence of *BMP2/4*, *BMP5-8*, and *GDF5-like* mRNA in the embryo at gastrulation (*34*, *35*) (Fig. 3B and fig. S4B). In light of this notable pro-BMP effect of *Nematostella* Chordin, it was important to verify that it can still act as a BMP antagonist, which we could confirm in our overexpression experiments both with the WT Chordin and with the membrane-tethered Chordin-CD2.

**Fig. 4.**
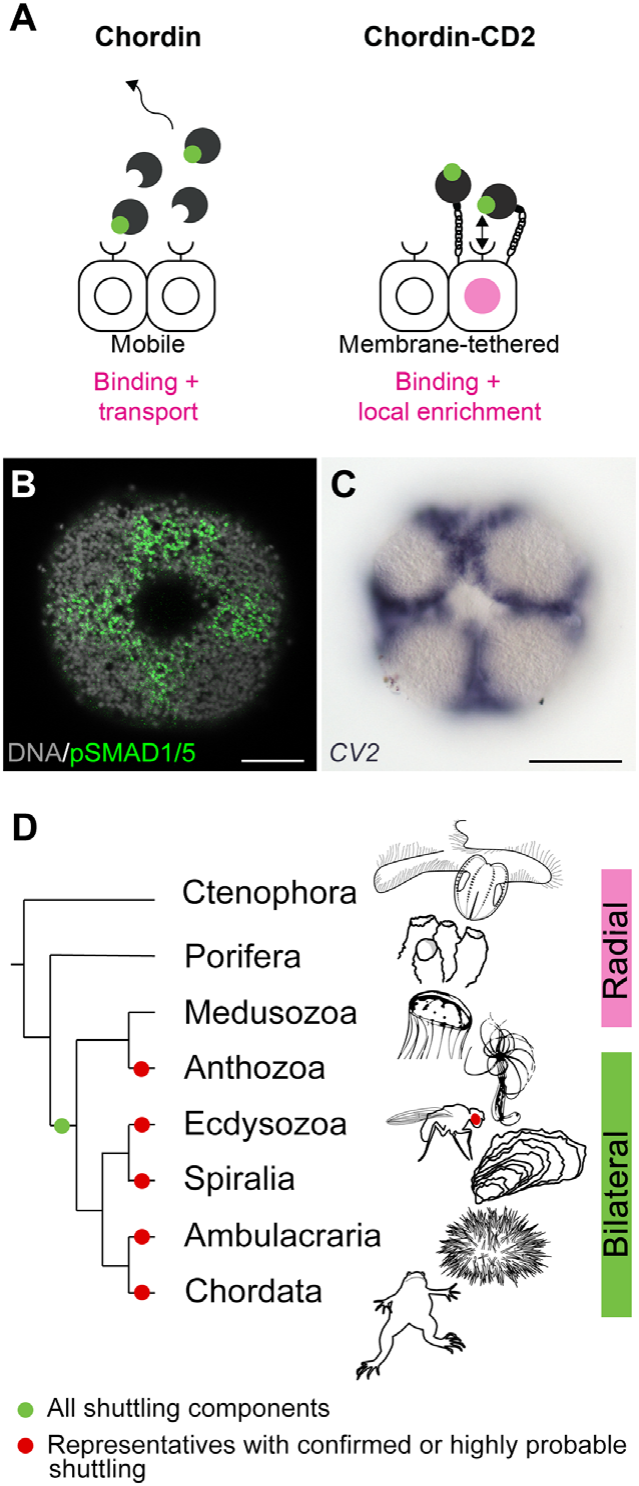
BMP shuttling-mediated bilaterality may have been present in the cnidarian-bilaterian ancestor. (**A**) BMP binding by the diffusible WT Chordin results in BMP ligand transport and local inhibition of BMP signaling. BMP binding by the membrane-tethered Chordin-CD2 results in a weak local activation of BMP signaling, potentially due to an enrichment of the BMP ligands close to the cell surface and a stimulation of the BMP-receptor interaction. (**B**) BMP signaling activity in the oral ectoderm of the 4-dpf late planula of *Nematostella*. pSMAD1/5 activity is observed between the future primary tentacle buds and around the mouth. (**C**) Expression of *Nematostella CV2* in the same domain. (**D**) Phylogenetic distribution of Chordin-mediated BMP shuttling components across Metazoa. Scale bars, [(B) and (C)] 50 μm.

Shuttling of BMP2/4 and BMP5-8 ligands by Chordin from the "low–BMP signaling" to the “high–BMP signaling” side of the directive axis provided a plausible explanation for the loss-of-function phenotypes observed in *Nematostella*; however, an alternative local inhibition mechanism, similar to the mechanism described in zebrafish, in which Chordin is simply a local BMP repressor, could not be excluded (*34*, *39*–*41*). To find out whether Chordin acts as a local BMP inhibitor or a long-range BMP shuttle during the directive axis formation in *Nematostella*, we established a localized source assay. We showed that mobile Chordin was required to promote strong BMP signaling at the side of the embryo opposite to the Chordin source, in line with the shuttling model. In contrast, not only was our membrane-tethered Chordin-CD2 unable to activate BMP signaling at a distance, but it activated it inside the Chordin-CD2 source instead. While this apparent local pro-BMP effect is surprising for a BMP inhibitor like Chordin, such behavior is not unheard of. Crossveinless-2 (CV2) is a BMP-binding protein with Chordin type cysteine-rich domains, which attaches to the cell surface by interacting with the side chains of heparan sulfate proteoglycans. Both in *Drosophila* and in vertebrates, CV2 is positively regulated by BMP signaling and exhibits complex pro- and anti-BMP effects (*56*–*59*). In *Nematostella*, a CV2 ortholog is not expressed in early embryos but becomes detectable in the pSMAD1/5-positive domain later in development (Fig. 4, A and C) (*60*). In *Drosophila*, the pro-BMP function of CV-2 was suggested to be due to it “handing over” sequestered BMPs to the type I BMP receptor Thickveins (*59*). We speculate that, by adding the CD2 sequence to the C terminus of Chordin, we may have created a CV2 analog. Its behavior indicates the direction for future research: It will be important to test experimentally whether WT, mobile Chordin facilitates the BMP-receptor interaction in 1- to 2-dpf *Nematostella* embryos. Should this be the case, this would explain the loss of BMP signaling in Chordin morphants despite the presence of BMPs in the embryo. On the other hand, in 4-dpf embryos, when the directive axis is already patterned, Chordin expression stops, and BMP signaling becomes confined to the areas where BMPs are expressed (*14*). Given the indispensability of Chordin for BMP signaling in the 1- to 2-dpf embryos, it will be interesting to find out how it is possible that Chordin becomes unnecessary during later stages. Based on its expression and the localization of BMP signaling activity, CV2 may be a good candidate for the role of “Chordin substitute” in the head ectoderm (*60*).

Chordin-dependent BMP shuttling has been experimentally demonstrated to be the mechanism responsible for the patterning of the dorsoventral body axis in a number of model organisms across bilaterian phyla (Fig. 4D). However, there are bilaterian clades in which the dorsoventral axis is patterned by other mechanisms (*2*). There is no proof that BMP shuttling represented the ancestral mode of the dorsoventral patterning in Bilateria, although we believe that this was the case because multiple instances of independent evolution of Chordin-dependent BMP shuttling on the very distant branches of the bilaterian tree involving the same molecular components appear less parsimonious than multiple independent losses of shuttling. Our work deepens this controversy even further. We showed that Chordin-dependent BMP shuttling described in Bilateria is also the mechanism responsible for the establishment and patterning the second body axis in the sea anemone *Nematostella*, a member of the bilaterian sister clade. Thus, the last common ancestor of Cnidaria and Bilateria had all the necessary components to establish a BMP shuttling-mediated symmetry break and maintain the second body axis (Fig. 4D). Whether this means that the cnidarian-bilaterian ancestor was a bilaterally symmetric animal, we cannot be certain, but it seems quite possible. On the other hand, the combination of BMP ligands and Chordin represents a powerful system to break symmetries and drive axial patterning, which in different animals has been assembled into such a variety of regulatory networks with different topologies, that convergent evolution of bilaterality in Cnidaria and Bilateria cannot be excluded.

## MATERIALS AND METHODS

### *Nematostella* culture

*N. vectensis* lab cultures were maintained and induced for spawning as previously described (*61*, *62*). Embryos and larvae were kept at 21°C, unless stated otherwise in the experimental procedures. No ethical committee approval is required for working with *Nematostella*, and no specific institutional guidelines exist other than the husbandry procedures developed by the Technau and Martindale labs in early 2000s.

### Fusion constructs, in vitro transcription, and transgenics

The sequences for expression constructs were generated using standard cloning techniques, the primers that were used are shown in table S1. Fusion constructs were generated via splicing by overlap extension polymerase chain reaction (*63*). In BMP2/4-FLAG-sfGFP, the FLAG-sfGFP tag is placed between the BMP2/4 pro-domain and the MLD after the predicted cleavage site RRKRSL (*64*) with LGDPPVAT linkers, mimicking previous zebrafish constructs (*39*). The BMP2/4 coding sequence in the construct contains four silent mutations to block binding of the previously tested BMP2/4MO (*9*). BMP2/4-mCherry is an equivalent fusion construct where mCherry replaces FLAG-sfGFP. proBMP2/4-FLAG-sfGFP corresponds to BMP2/4-FLAG-sfGFP with the MLD removed. Chordin-FLAG-sfGFP encodes a protein where FLAG-sfGFP is fused to the C terminus of Chordin. BMP5-8–mCherry and GDF5L-mCherry contain the mCherry sequence flanked by LGDPPVAT linkers at the beginning of their mature domains, following the predicted cleavage sites RVSRSL and QREKRK, respectively. For Chordin-CD2 and Chordin-FLAG-sfGFP-CD2, the CD2 sequence (*32*) is fused to the C terminus of Chordin and Chordin-FLAG-sfGFP, respectively. mRNA coding for mCherry with the C-terminal CAAX sequence (*65*) was used to label the mRNA injected cells in the localized source experiments. Sequences for mRNA synthesis were cloned downstream of the T7 promoter into a customized pJet1.2 plasmid (Thermo Fisher Scientific) carrying Pac I and Sbf I sites followed by the SV40 polyadenylation signal. The mMessage mMachine T7 Transcription Kit (Thermo Fisher Scientific) was used for in vitro transcription. To generate the transgenic BMP2/4::BMP2/4-FLAG-sfGFP line, a 4.5-kb DNA fragment upstream of the translation start site of the *BMP2/4* gene (*42*) was cloned into a previously described transgenesis vector (*66*) to drive the expression of *BMP2/4-FLAG-sfGFP*. Plasmid (50 ng/μl) was digested with 0.2 U of I–Sce I/μl for 30 min at 37°C before injection. For live imaging of the *BMP2/4::BMP2/4-FLAG-sfGFP* embryos, an F0 transgenic male was crossed to a WT female, and the embryos were injected at the one-cell stage with *mCherry-CAAX* mRNA (61 ng/μl). GFP-positive embryos were mounted in 2% low-melting agarose in *Nematostella* medium on a cover slip. Upon solidification of the agarose, the cover slip was placed in a petri dish lid, covered with *Nematostella* medium and imaged on a Leica TCS SP8 confocal microscope using a 40× water-dipping objective.

### Gene knockdown, overexpression, and the local source assay

Previously characterized morpholino oligonucleotides against *Chd*, *BMP2/4*, and *GDF5-like* (*9*, *34*) were injected into dejellied *Nematostella* zygotes as described in (*66*). A splice MO targeting exon 9 of *Mef2* was used as a Control MO as in (*67*), because it does not affect development at least until primary polyp stage and shows lower toxicity than the Gene Tools standard control MO. All MO sequences can be found in table S2. For overexpression, mRNAs were injected into zygotes at concentrations summarized in table S3. All injection mixes contained Dextran–Alexa Fluor 568 (30 ng/μl; Invitrogen) as a tracer. For the local source assay, *Nematostella* zygotes were injected with the splice morpholino against Chordin (ChdMO) with or without BMP2/4MO and GDF5lMO and kept at 18.5°C until they started dividing. When the embryos reached eight-cell stage, individual blastomeres were injected with mRNA mixtures described in table S3. The embryos were kept at 21°C and fixed at 1 dpf [24 to 26 hours postfertilization (hpf)] for anti-pSMAD1/5 and anti-mCherry immunostaining as described below.

### Immunohistochemistry and in situ hybridization

For immunohistochemistry and in situ hybridization, the embryos were fixed in ice-cold 0.25% glutaraldehyde/3.7% formaldehyde/PTx (PTx = 1× PBS with 0.3% Triton X-100) for 2 min on ice and then in 3.7% formaldehyde/PTx for 1 hour at 4°C with overhead rotation. For immunohistochemistry, fixed embryos were washed five times for 5 min in PTx and then incubated in prechilled methanol on ice for 8 min, washed three more times with PTx, and blocked in a blocking solution containing 5% heat-inactivated sheep serum and 95% of 1% BSA/PTx for 2 hours at room temperature. At the same time, primary antibodies were diluted and preincubated in the blocking solution. The embryos were stained overnight with rabbit monoclonal anti-pSMAD1/5/9 (Cell Signaling Technology, 13820) in 1:200 dilution and mouse monoclonal anti-mCherry antibody (Takara, 632543) in a 1:400 dilution at 4°C on rocker. After five 15-min PTx washes, the embryos were blocked again, and primary antibodies were detected with goat anti-rabbit Alexa Fluor 488 (Invitrogen, A-11008) and goat anti-mouse Alexa Fluor 633 (Invitrogen, A-21050) antibodies in a 1:1000 dilution for 2 hours at room temperature on the rocker. 4′,6-Diamidino-2-phenylindole (5 μg/ml) was added to the secondary antibody solution to make sure that pSMAD1/5 signal is nuclear. sfGFP fluorescence was visible after fixation, and no additional antibody staining was performed; Alexa Fluor 633–phalloidin (Invitrogen, A22284) was used at a final concentration of 4 U/ml. The embryos were imaged with a Leica SP8 or Leica Stellaris 5 laser-scanning confocal microscope.

For in situ hybridization, the embryos were fixed as described above, washed five times in PTx or PTw (PTw = 1× PBS with 0.1% Tween 20) and once in 100% methanol, and stored in 100% methanol at −20°C. After rehydration by washing for 5 min in 50% methanol/PTw and in pure PTw, the embryos were handled as described in (*14*) with the following change: Proteinase K treatment was performed with proteinase K/PTw solution (10 μg/ml) for 20 min.

### Embryo fractionation to sample secreted proteins

Embryos were injected with the different combinations of mRNAs (equimolar between samples) and incubated at 21°C until the cells started dividing. One hundred dividing embryos of a sample were transferred to a well of a 96-well plate in 90 μl of *Nematostella* medium and incubated at 21°C overnight. Around 22 hpf, the embryos and the medium were transferred to a 1.5-ml reaction tube. Eighty microliters of medium was removed and mixed with 25 μl of 5× loading dye as “medium” fraction. Eighty microliters of Mg^2+^/Ca^2+^-free artificial sea water [NaCl (27 g/liter), Na_2_SO_4_ (1 g/liter), KCI (0.8 g/liter), and NaHCO_3_ (0.18 g/liter); (*43*)] containing cOmplete Protease Inhibitor Cocktail (Roche) was added to the embryos, and embryos were dissociated into a cell suspension by trituration. After 1 min of centrifugation at 1000 rcf at room temperature, 80 μl of supernatant was mixed with 25 μl of 5× gel loading dye as EC (extracellular) fraction. The cells were then lysed in 80 μl of cold Cell Extraction Buffer (Life Technologies/Invitrogen) containing cOmplete Protease Inhibitor Cocktail (Roche). After 10 min of centrifugation at 16,000 rcf (4°C), 80 μl of supernatant was collected and mixed with 25 μl of 5× gel loading dye as cell fraction.

### Co-immunoprecipitation

Embryos were injected with a mix of mRNAs for mCherry-tagged proteins (*BMP2/4-mCherry*, *BMP5-8–mCherry*, and *GDF5L-mCherry*) and bait proteins (*BMP2/4-FLAG-sfGFP*, *Chordin-FLAG-sfGFP*, and *Chordin-FLAG-sfGFP-CD2*) or *GFP* mRNA (negative control). At 1 dpf (~21 hpf), ~230 embryos per sample were dissociated in 130 μl of Mg^2+^/Ca^2+^-free artificial sea water (with protease inhibitors) to prepare EC protein fractions as described above. The cells were lysed in 130 μl of lysis buffer [10 mM tris, 150 mM NaCl, 0.5 mM EDTA, and 0.5% NP-40 substitute (Merck) (pH 7.5)] containing cOmplete Protease Inhibitor Cocktail (Roche). For the IP, GFP-Trap Magnetic Agarose beads (Chromotek) were used according to the manufacturer’s recommendations. Per IP, 12.5 μl of agarose slurry was used and equilibrated by rinsing with 500 μl of dilution buffer [10 mM tris, 150 mM NaCl, and 0.5 mM EDTA (pH 7.5)] three times. The agarose was suspended in dilution buffer for IPs from cell lysates and in a 2:1 lysis buffer:dilution buffer mix for IPs from EC fractions and supplemented with cOmplete Protease Inhibitor Cocktail (Roche). Of the 130-μl EC fraction/cell lysate, 6 μl was saved as 5% input and 120 μl was mixed with 180 μl of beads prepared for the IP as described above (10% input was collected for the GDF5L-mCherry containing IPs). During IP, the tubes were incubated at 4°C for 1 hour with overhead rotation. The beads were then washed three times for 5 min with 500 μl of wash buffer [10 mM tris, 150 mM NaCl, 0.5 mM EDTA, and 0.05% NP-40 substitute (Merck) (pH 7.5)] containing cOmplete Protease Inhibitor Cocktail (Roche) and transferred to a fresh tube during the last wash step. To collect the IP samples, the beads were heated to 95°C in 50 μl of 1× protein loading buffer for 5 min.

### SDS–polyacrylamide gel electrophoresis and Western blot

Whole-embryo protein lysates were prepared with the Cell Extraction Buffer (Life Technologies/Invitrogen) containing cOmplete Protease Inhibitor Cocktail (Roche) that was additionally supplemented with PhosSTOP Phosphatase Inhibitor Cocktail (Roche) if the aim was to detect pSMAD1/5. Proteins were separated on 10% polyacrylamide gels and blotted onto a nitrocellulose membrane at 100 V for 1 hour using the Mini Trans-Blot system (Bio-Rad). Membranes were blocked with 5% milk powder in PTw (1× PBS and 0.1%Tween 20), and the same blocking solution was used for the following antibody dilutions: 1:10,000 anti-GFP (Abcam ab290), 1:2000 monoclonal anti-mCherry (Takara/Clontech, no. 632543), 1:1000 polyclonal anti-mCherry (Chromotek pabr1; used only for the detection of BMP2/4-mCherry in CoIP experiments), 1:10,000 anti–β-actin (Cell Signaling Technology, no. 4970), and 1:10,000 of the horseradish peroxidase (HRP)–conjugated anti-rabbit immunoglobulin G (IgG; Promega W401B) and anti-mouse IgG (Promega W402B). For the pSMAD1/5 Western blot, the protocol of (*68*) was followed using 1:1000 anti–phospho-SMAD1/SMAD5/SMAD9 (Cell Signaling Technology, no. 11971) and 1:10,000 HRP-conjugated anti-rabbit IgG (Promega, W401B). The SuperSignal West Femto Maximum Sensitivity Substrate (Thermo Fisher Scientific) was used for enhanced chemiluminescence detection. Western blot band intensities were quantified in Fiji (*69*) using rectangular regions of interest. Background intensities were subtracted, and the background-subtracted intensities were normalized as described.
