# Supplementary material for: Chordin-mediated BMP shuttling patterns the secondary body axis in a cnidarian
Source: Sci Adv. 2025 Jun 13;11(24):eadu6347. doi: 10.1126/sciadv.adu6347 (PMC12164971; doi:10.1126/sciadv.adu6347)
Supplement: Supplementary file 1 — Figs. S1 to S5 Tables S1 to S3 [file sciadv.adu6347_sm.pdf]

Science Advances
AAAS

Supplementary Materials for

Chordin-mediated BMP shuttling patterns the secondary body axis in a cnidarian

David Mörsdorf *et al.*

Corresponding author: Grigory Genikhovich, grigory.genikhovich@univie.ac.at

Sci. Adv. **11**, eadu6347 (2025)
DOI: 10.1126/sciadv.adu6347

This PDF file includes:

Figs. S1 to S5
Tables S1 to S3

Fig. S1.

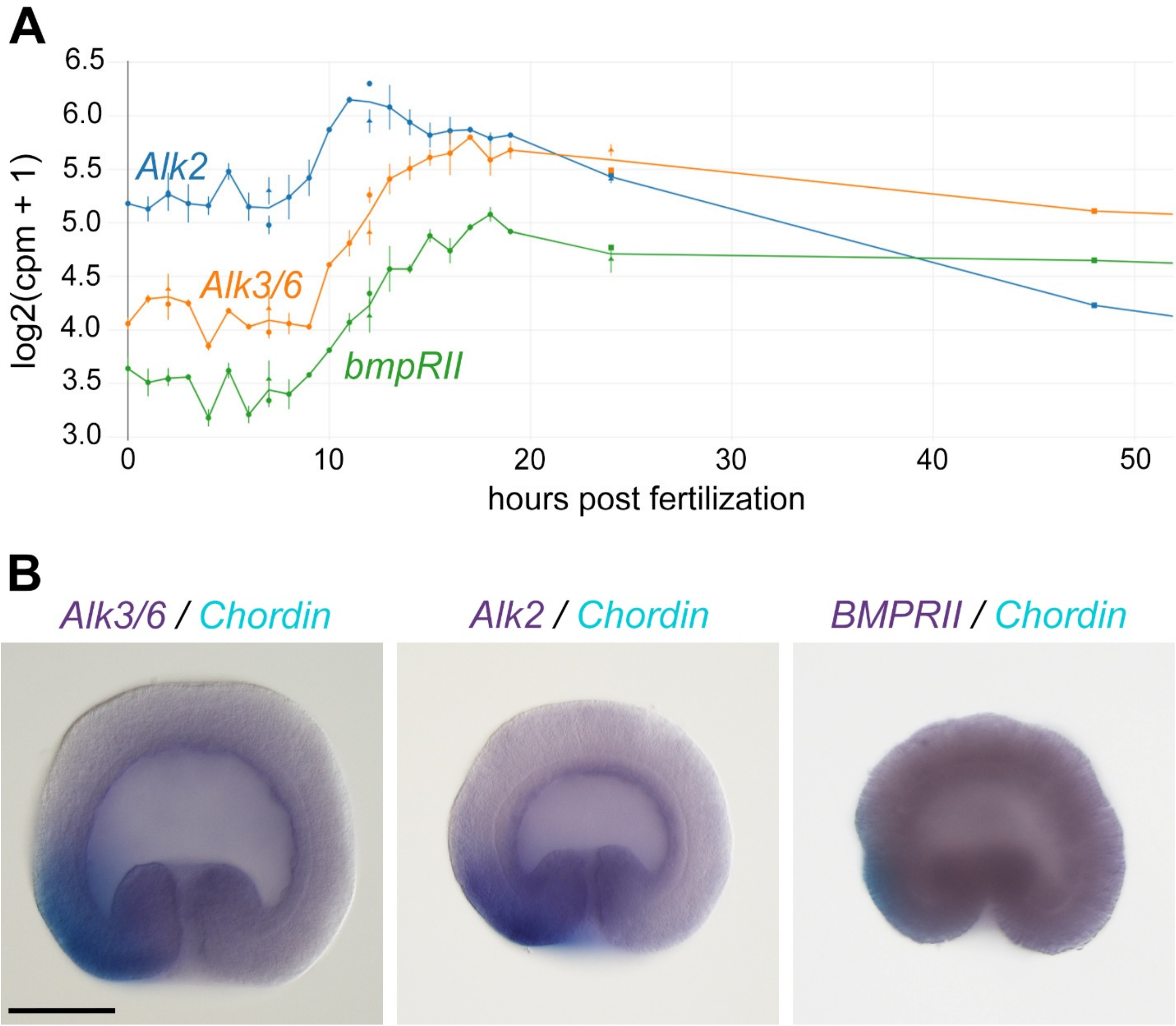

Figure S1. Normal expression of the BMP receptors in the early embryo of *Nematostella*. A) RNA-Seq based expression plot for the type I BMP receptors *Alk2* and *Alk3/6* and of the type II BMP receptor *BMPRII* during the first 52 hours of *Nematostella* development. Screenshot from the NvERTx expression database (https://nvertx.ircan.org/ER/ER_plotter/home , (*36*)). **B)** Double in situ hybridization of the late gastrula stage embryos shows homogenous expression of the BMP receptors at this stage (purple staining). *Chordin* probe stains the "low pSMAD1/5" side of the newly formed directive axis (blue staining on the left side of each embryo). Blastopores point down. Scale bar 100 µm.

Fig. S2.

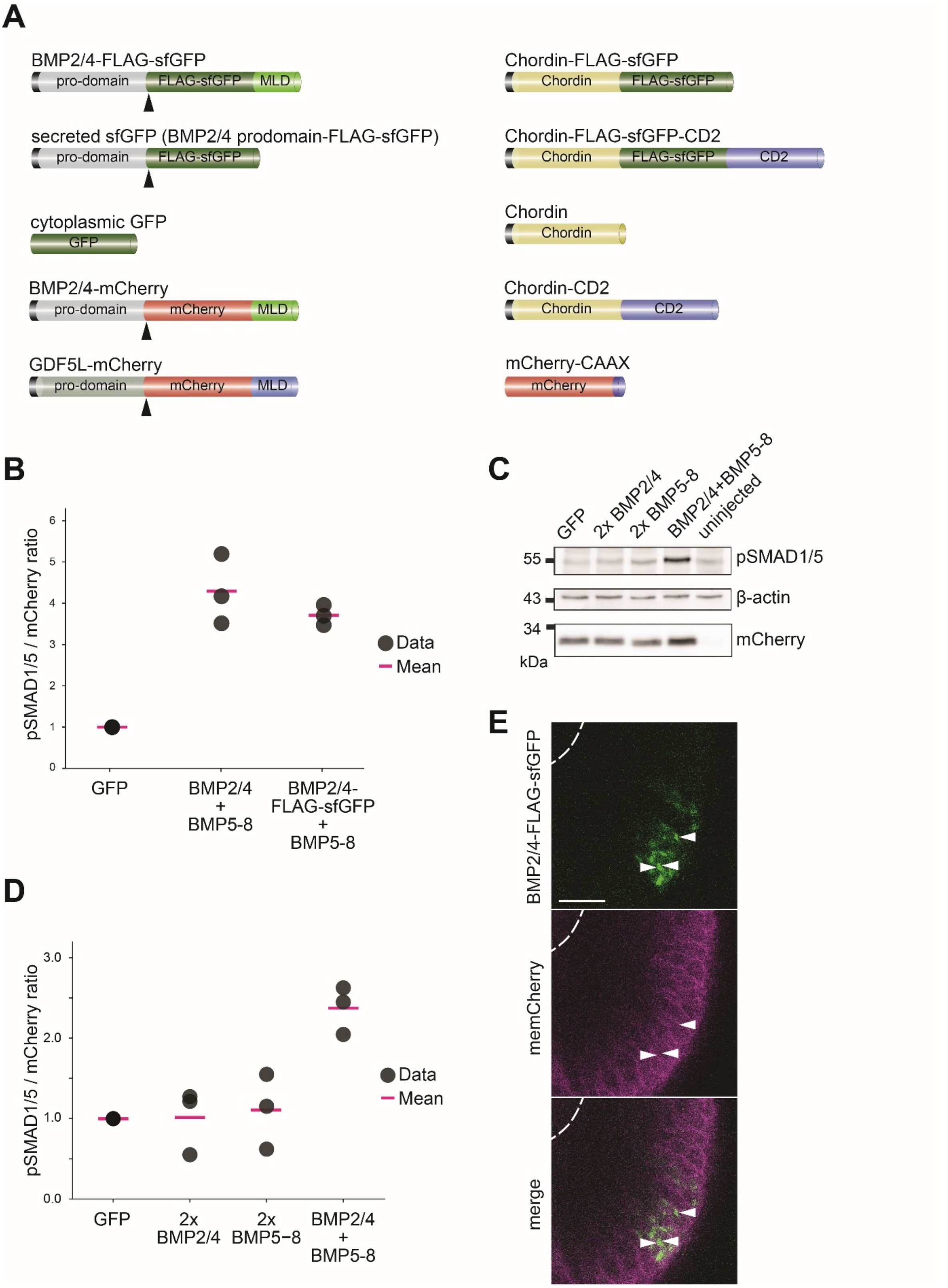
A
BMP2/4-FLAG-sfGFP
pro-domain
FLAG-sfGFP
MLD
secreted sfGFP (BMP2/4 prodomain-FLAG-sfGFP)
cytoplasmic GFP
GFP
BMP2/4-mCherry
mCherry
GDF5L-mCherry
Chordin-FLAG-sfGFP
Chordin
Chordin-FLAG-sfGFP-CD2
CD2
Chordin
Chordin-CD2
mCherry-CAAX
B
pSMAD1/5 / mCherry ratio
Data
Mean
GFP
BMP2/4 + BMP5-8
BMP2/4-FLAG-sfGFP + BMP5-8
C
GFP
2x BMP2/4
2x BMP5-8
BMP2/4+BMP5-8
uninjected
55
43
34
kDa
pSMAD1/5
β-actin
mCherry
D
pSMAD1/5 / mCherry ratio
Data
Mean
GFP
2x BMP2/4
2x BMP5−8
BMP2/4 + BMP5-8
E
BMP2/4-FLAG-sfGFP
memCherry
merge

Figure S2. BMP2/4-FLAG-sfGFP is an active ligand eliciting stronger pSMAD1/5 signal in combination with BMP5-8. A) Schemes of constructs used in the paper. Black arrowhead demarcates the cleavage site separating the prodomain from the mature ligand domain (MLD). **B)** Quantification of the western blot shown in Fig. 2A and two additional independent replicates. After quantification, the pSMAD1/5-to-mCherry ratio was normalized to the GFP sample. *N*=3. **C)** Western blot shows that co-injection of *BMP2/4* and *BMP5-8* mRNAs elicits a stronger pSMAD1/5 signal than each mRNA alone injected at double the concentration (labelled as 2x). mCherry signal was used as an injection reference. **D)** Quantification of data in (C) and two additional, independent replicates. *N*=3 **E)** Live imaging of 1 dpf *BMP2/4::BMP2/4-FLAG-sfGFP* embryos injected with *mCherry-CAAX* mRNA (memCherry) at the one-cell stage show GFP signal inside the ectodermal cells at their apical side (white arrowheads). White dashed line demarcates the basal boundary of the ectodermal cells where they attach to the mesoglea. *N*=3. Scale bar 20 μm.

Fig. S3.

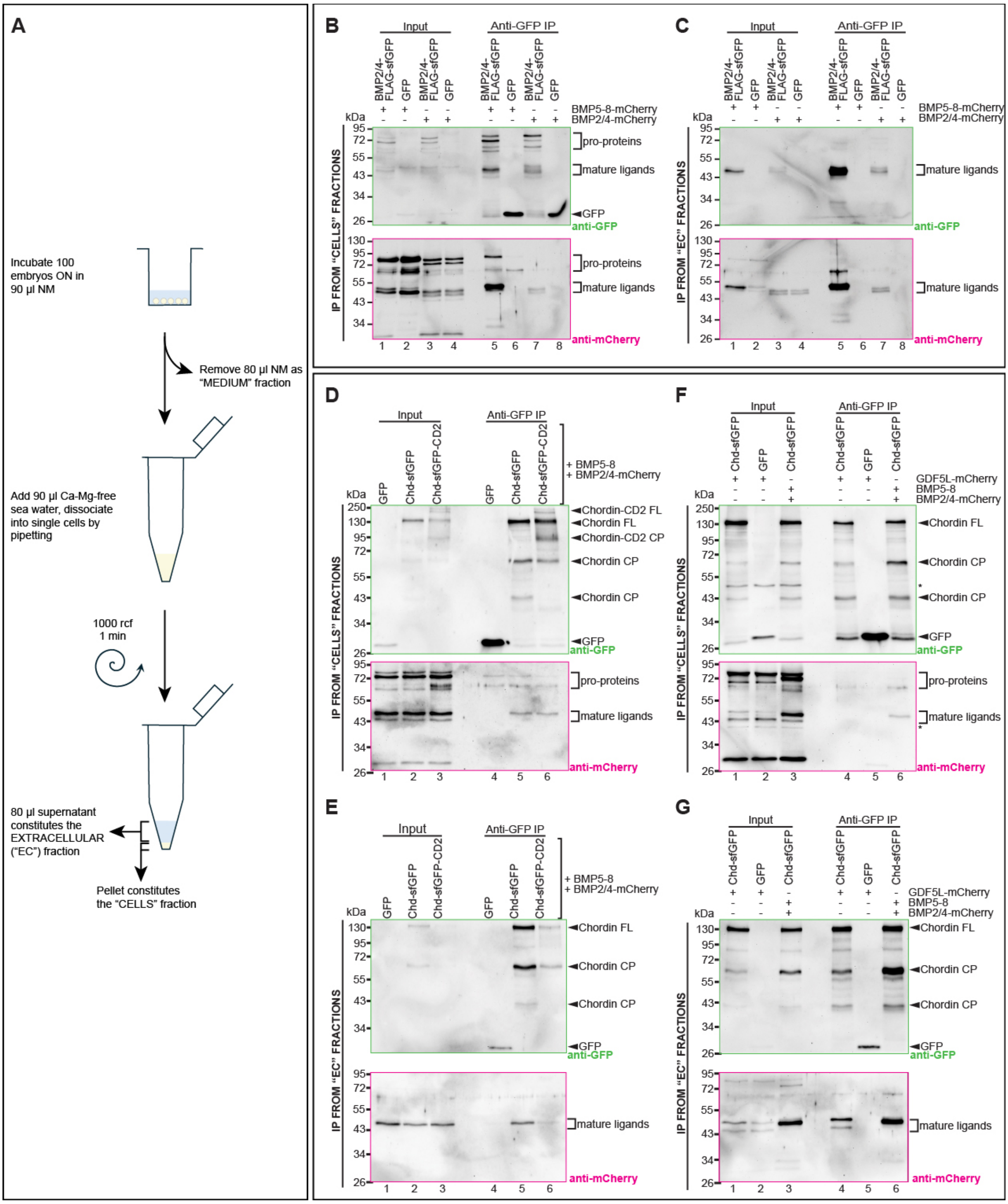

Figure S3. CoIP confirms that BMP2/4 and BMP5-8 can form a heterodimer and that Chordin can form an extracellular complex with BMP2/4 as well as with GDF5L. A) Fractionation scheme used to obtain proteins released into the medium, associated with the cell surface, and located in the cellular fraction. **B)** Anti-GFP CoIP from cellular protein fractions of

embryos expressing either BMP5-8-mCherry or BMP2/4-mCherry as well as BMP2/4-FLAG-sfGFP or GFP (control). Mature BMP5-8-mCherry (compare lanes 5 and 6) and, to a lesser extent, mature BMP2/4-mCherry (compare lanes 7 and 8) co-immunoprecipitate with BMP2/4-FLAG-sfGFP revealing the presence of both hetero- and homodimers. *N*=2. **C)** Anti-GFP CoIP from extracellular protein fractions of the same embryos used in B). Homo-and heterodimers are detected also in the extracellular fraction (compare lanes 5 with 6 and lanes 7 with 8). *N*=2. **D)** Anti-GFP CoIP from cellular protein fractions of embryos expressing both BMP5-8 and BMP2/4-mCherry as well as Chordin or GFP (control) bait proteins. BMP2/4-mCherry co-immunoprecipitates with both Chordin-FLAG-sfGFP (Chd-sfGFP; compare lanes 4 and 5) and Chordin-FLAG-sfGFP-CD2 (Chd-sfGFP-CD2; compare lanes 4 and 6). *N*=3. **E)** Anti-GFP CoIP from extracellular protein fractions of the same embryos used in D). BMP2/4-mCherry co-immunoprecipitates with Chordin-FLAG-sfGFP (Chd-sfGFP; compare lanes 4 and 5), revealing the presence of an extracellular BMP-Chordin complex. *N*=3. **F)** Anti-GFP CoIP from cellular protein fractions of embryos expressing either GDF5L-mCherry or both BMP5-8 and BMP2/4-mCherry together with Chordin-sfGFP or GFP (control). *N*=2. **G)** Anti-GFP CoIP from extracellular protein fractions of the same embryos used in F). Not only mature BMP2/4-mCherry, but also mature GDF5L-mCherry co-immunoprecipitates with Chordin-FLAG-sfGFP (Chd-sfGFP; compare lane 4 with lanes 5 and 6). *N*=2.

Fig. S4.

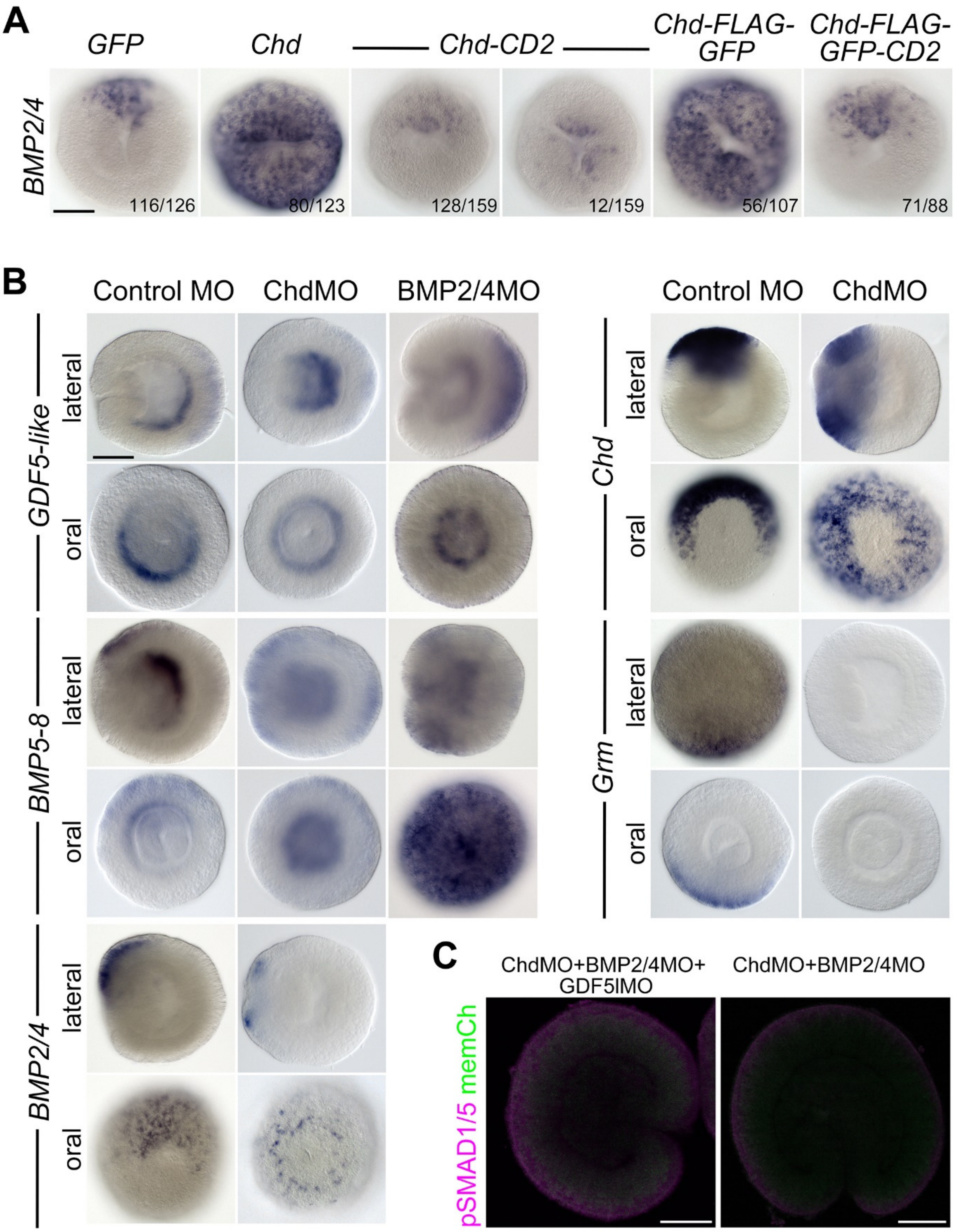
A
GFP
Chd
Chd-CD2
Chd-FLAG-GFP
Chd-FLAG-GFP-CD2
BMP2/4
116/126
80/123
128/159
12/159
56/107
71/88
B
Control MO
ChdMO
BMP2/4MO
Control MO
ChdMO
GDF5-like
lateral
oral
BMP5-8
lateral
oral
BMP2/4
lateral
oral
Chd
lateral
oral
Grm
lateral
oral
C
ChdMO+BMP2/4MO+GDF5lMO
ChdMO+BMP2/4MO
pSMAD1/5 memCh

Figure S4. Chordin can act as a BMP inhibitor, and upon its knockdown, the mRNA of BMP ligands is present in 1 dpf embryo. A) mRNA injection followed by *BMP2/4 in situ* hybridization at 30 hpf shows that Chordin and Chordin-FLAG-sfGFP repress BMP signaling, resulting in radialized *BMP2/4* expression. *Chordin-CD2* and *Chordin-FLAG-sfGFP-CD2* mRNA injections result in a mostly asymmetric expression of *BMP2/4*, indicating the presence of a BMP signaling gradient. Oral views. Numbers in the bottom right corner show the fraction of the embryos demonstrating the phenotype shown on the representative image. *N*=2. **B)** *In situ* hybridization after the injection of ChdMO shows that the BMP ligands *BMP2/4*, *BMP5-8* and *GDF5-like*, as well as *Chd* are expressed in a radially symmetric manner at 1 dpf, whereas the expression of the direct BMP signaling target *Grm* is lost. *BMP5-8* and *GDF5-like* mRNA is present in a radially symmetric domains in embryos injected with BMP2/4MO. n > 50 for each knockdown experiment. On lateral views blastopores point to the left. *N*=3. **C)** Similar to ChdMO injection, combined knockdown of Chd, BMP2/4 and GDF5-like or Chd and BMP2/4 completely abolishes BMP signaling activity. *N*=3. Scale bars 50 µm.

Fig. S5.

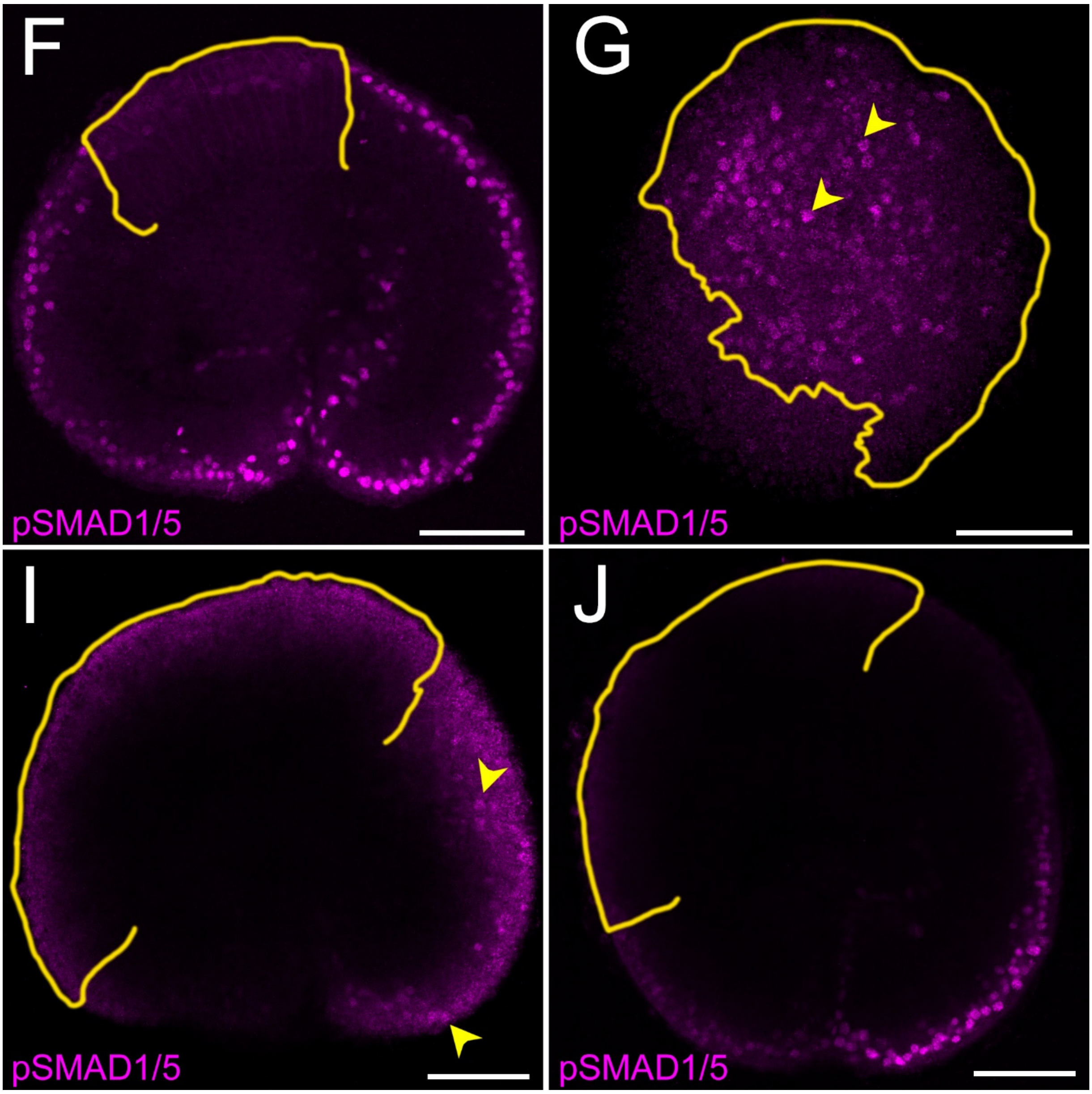

Figure S5. Images F, G, I, and J from the main text Fig. 3 shown without the green channel. F) ChdMO injected embryo with a source of wild type Chordin in the area marked with the yellow line shows maximum pSMAD1/5 signal on the side opposite to the Chd source. **G)** ChdMO injected embryo with a source of Chd-CD2 marked with the yellow line shows pSMAD1/5 within the Chd-CD2 source. **I)** ChdMO+BMP2/4MO+GDF5-likeMO injected embryo with a Chd, BMP2/4 and BMP5-8 source marked with the yellow line shows pSMAD1/5 signal on the side opposite to the Chd/BMP source. **J)** ChdMO+BMP2/4MO injected embryo with a Chd source marked with the yellow line shows pSMAD1/5 signal on the side opposite to the Chd/BMP source. Yellow arrowheads point at the same nuclei as on the main text Fig. 3. Scale bars 50 µm.

Table S1. Primers used in this study.

Name	Sequence
BMP2/4 MO mismatch PacI F	GTTAATTAAATGCTGCTCCTACGGTGCTTCTTACTACTG
BMP2/4 SbfI R	CTAGAGTCCCTGCAGGCTACCTACAGCCGCAC
BMP2/4-FLAG-sfGFP splice R1	TCGTCATCGTCCTTGTAATCGGTGGCGACCGGTGG
BMP2/4-FLAG-sfGFP splice F1	ACAAGGACGATGACGATAAGGTGTCTAAAGGAGAG
BMP2/4-FLAG-sfGFP splice R2	CGCCGGACCGTGTCGCCACTGGCGGGTCTCCAAGTTTGTACAGCTCATC
BMP2/4-FLAG-sfGFP splice F2	GTGGCGACACGGTCCGGCGGCGCCAAAAG
BMP2/4-mCherry splice R1	CTCGCCCTTGCTCACGGTGGCGACCGGTG
BMP2/4-mCherry splice F1	CACCGGTCGCCACCGTGAGCAAGGGCGAG
BMP2/4-mCherry splice R2	CTGGCGGGTCTCCAAGCTTGTACAGCTCGTC
BMP2/4-mCherry splice F2	GACGAGCTGTACAAGCTTGGAGACCCGCCAG
BMP5-8 PacI F	GTAATTTAATTAAATGCGGAGAATACGAGACCTG
BMP5-8 SbfI R	CTAGAGTCCCTGCAGGCTAGTGACATCCACAAG
BMP5-8-mCherry splice F1	CTGGGCGATCCACCGGTCGCCACCGTGAGCAAGGGCGAG
BMP5-8-mCherry splice R1	GGTGGCGACCGGTGGATCGCCCAGTAGCGACCTGGAGAC
BMP5-8-mCherry splice F2	CTTGGAGACCCGCCAGTGGCGACACCGAACGCACAGGTG
BMP5-8-mCherry splice R2	GTGTCGCCACTGGCGGGTCTCCAAGCTTGTACAGCTCGTC
Chd PacI F	CAGGGTAATTTAATTAAATGTTGGCCAAATTC
Chd SbfI R	CTAGAGTCCCTGCAGGTTACCCCGAGCAAGG
Chd-CD2 splice R	GTACCGCGATATCAGCCCCCGAGCAAGGAACAC
Chd-CD2 splice F	GTGTTCCTTGCTCGGGGGCTGATATCGCGGTAC
CD2 SbfI R	GCACCTGCAGGTTAATTAGGGGGTGGCAAC
Chd-FLAG-sfGFP splice F	GTTCCTTGCTCGGGGGATTACAAGGACGATG
Chd-FLAG-sfGFP splice R	CATCGTCCTTGTAATCCCCCGAGCAAGGAAC
Chd-FLAG-sfGFP SbfI R	CACCTGCAGGTTATTTGTACAGCTCATCC
sfGFP-CD2 splice R	GTACCGCGATATCAGCTTTGTACAGCTCATCC
sfGFP-CD2 splice F	GGATGAGCTGTACAAAGCTGATATCGCGGTAC
mCherry-CAAX PacI F	GTTAATTAAATGGTGAGCAAGGGCGAGGAGG
mCherry-CAAX SbfI R	CACCTGCAGGTCAGGAGAGCACAC

GDF5L PacI F	GCAAGTTAATTAAATGCAAGCGTGGTTTAGG
GDF5L SbfI R	CACCTGCAGGTTAACTGCACCCACATC
GDF5L-mCherry splice F1	GAGAAAAGCGCAAACTGGGCGATCCACCG
GDF5L-mCherry splice R1	CGGTGGATCGCCCAGTTTGCGCTTTTCTC
GDF5L-mCherry splice F2	CCGCCAGTGGCGACATGCAGACGAAAAAGAATG
GDF5L-mCherry splice R2	CATTCTTTTTCGTCTGCATGTCGCCACTGGCGG

Table S2. Morpholino oligonucleotides used in this study.

Name	Sequence
BMP2/4MO	GTAAGAAACAGCGTAAGAGAAGCAT
ChdMO	GATCCACTCACCATCTTTGCGAGAC
GDF5-likeMO	AGGTTATTTAGCCTGACCTTGATCG
Control MO	ATGTGCCTAGGGTACAACAACAAT

Table S3. Details on concentrations used in the injection mixtures.

Experiment	Sample	Component	Final conc.
**BMP2/4-FLAG-sfGFP function**	BMP2/4	*BMP2/4* mRNA	100 ng/µl
		BMP 5-8 mRNA	100 ng/µl
		mCherry-CAAX mRNA	70 ng/µl
	BMP2/4-FLAG-sfGFP	*BMP2/4-FLAG-sfGFP* mRNA	160 ng/µl
		BMP 5-8 mRNA	100 ng/µl
		mCherry-CAAX mRNA	70 ng/µl
	GFP	*GFP* mRNA	140 ng/µl
		mCherry-CAAX mRNA	70 ng/µl
**secretion/fractionation**	BMP2/4-FLAG-sfGFP	*BMP2/4-FLAG-sfGFP* mRNA	100 ng/µl
	proBMP2/4-FLAG-sfGFP	*proBMP2/4-FLAG-sfGFP* mRNA	100 ng/µl
	GFP	*GFP* mRNA	100 ng/µl
**secretion/fractionation (+BMP5-8)**	BMP2/4-FLAG-sfGFP	*BMP2/4-FLAG-sfGFP* mRNA	111 ng/ul
		BMP 5-8 mRNA	70 ng/µl
**BMP heterodimer signaling**	BMP2/4	*BMP2/4* mRNA	200 ng/µl
		mCherry-CAAX mRNA	70 ng/µl
	BMP5-8	*BMP 5-8* mRNA	200 ng/µl
		mCherry-CAAX mRNA	70 ng/µl
	BMP2/4 + BMP5-8	*BMP2/4* mRNA	100 ng/µl
		BMP 5-8 mRNA	100 ng/µl
		mCherry-CAAX mRNA	70 ng/µl
	GFP	*GFP* mRNA	140 ng/µl
		mCherry-CAAX mRNA	70 ng/µl
**Chordin-CD2 function**	Chordin	*Chordin* mRNA	170 ng/µl
	Chordin-CD2	*Chordin-CD2* mRNA	230 ng/µl
	GFP	*GFP* mRNA	58 ng/µl
	Chordin-FLAG-sfGFP	*Chordin-FLAG-sfGFP* mRNA	215 ng/µl
	Chordin-FLAG-sfGFP-CD2	*Chordin-FLAG-sfGFP-CD2* mRNA	275 ng/µl

**Single-blastomere injections (ChdMO)**	Chordin	*Chordin* mRNA<br>*mCherry-CAAX* mRNA	170 ng/µl<br>70 ng/µl
	Chordin-CD2	*Chordin-CD2* mRNA<br>*mCherry-CAAX* mRNA	230 ng/µl<br>70 ng/µl
**Single-blastomere injections (ChdMO + BMP2/4MO + GDF5-likeMO)**	Chordin	*Chordin* mRNA<br>*mCherry-CAAX* mRNA	170 ng/µl<br>70 ng/µl
	Chordin + BMPs	*Chordin* mRNA<br>*mCherry-CAAX* mRNA<br>*BMP2/4* mRNA<br>*BMP 5-8* mRNA	170 ng/µl<br>70 ng/µl<br>70 ng/µl<br>70 ng/µl
**Chordin-BMP CoIP**	GFP	*BMP 5-8* mRNA<br>*BMP2/4-mCherry* mRNA<br>*GFP* mRNA	70 ng/µl<br>109 ng/µl<br>116 ng/µl
	Chordin	*BMP 5-8* mRNA<br>*BMP2/4-mCherry* mRNA<br>*Chordin-FLAG-sfGFP* mRNA	70 ng/µl<br>109 ng/µl<br>430 ng/µl
	Chordin-CD2	*BMP 5-8* mRNA<br>*BMP2/4-mCherry* mRNA<br>*Chordin-FLAG-sfGFP-CD2* mRNA	70 ng/µl<br>109 ng/µl<br>550 ng/µl
**BMP dimer CoIP**	BMP2/4 + BMP5-8	*BMP2/4-FLAG-sfGFP* mRNA<br>*BMP5-8-mCherry* mRNA	111 ng/µl<br>112 ng/µl
	GFP + BMP5-8	*GFP* mRNA<br>*BMP5-8-mCherry* mRNA	49 ng/µl<br>112 ng/µl
	BMP2/4 + BMP2/4	*BMP2/4-FLAG-sfGFP* mRNA<br>*BMP2/4-mCherry* mRNA	111 ng/µl<br>109 ng/µl
	GFP + BMP2/4	*GFP* mRNA<br>*BMP2/4-mCherry* mRNA	49 ng/µl<br>109 ng/µl

**Chordin-GDF5L CoIP**	GFP	*GDF5L-mCherry* mRNA	236 ng/µl
		GFP mRNA	116 ng/µl
	BMPs	*BMP 5-8* mRNA	70 ng/µl
		BMP2/4-mCherry mRNA	109 ng/µl
		Chordin-FLAG-sfGFP mRNA	430 ng/µl
	GDF5L	*GDF5L-mCherry* mRNA	236 ng/µl
		Chordin-FLAG-sfGFP mRNA	430 ng/µl
